# From stress to cognitive impairment: an integrative model of oxidative and hemorheological mechanisms

**DOI:** 10.1590/1980-5764-DN-2025-0466

**Published:** 2026-09-14

**Authors:** Marcel Valério de Arruda, Marcelo Bussotti Reyes, Ruth Ferreira Galduróz

**Affiliations:** 1 Universidade Federal do ABC, Centro de Matemática, Computação e Cognição, Programa de Pós-Graduação em Neurociência e Cognição, São Bernardo do Campo, SP, Brazil.

**Keywords:** Psychological Stress, Cognition, Oxidative Stress, Nitric Oxide, Hemorheology, Estresse Psicológico, Cognição, Estresse Oxidativo, Óxido Nítrico, Hemorreologia

## Abstract

Psychological stress is associated with oxidative stress, which can have profound implications for cognitive functions. The Free Radical Theory of Aging highlights the potential role of accumulated oxidative damage in cellular aging and the development of age-related diseases. This imbalance is linked to alterations in erythrocyte rheology, potentially contributing to reduced blood flow and impaired cardiovascular function. Due to its high metabolic activity and sensitivity to oxidative conditions, the brain is particularly vulnerable to these effects. An integrative pathway is proposed that describes a cascade of interactions from psychological stress to reduced cognitive function. In this model, psychological stress is hypothesized to contribute to hemorheological changes that decrease the availability of oxygen and nutrients to neural tissues, subsequently impacting cognitive performance. Finally, this manuscript is a narrative review that presents a conceptual framework summarized in an integrative figure, highlighting the interplay between oxidative, vascular, and hemorheological mechanisms.

## RESUMO

O estresse psicológico está associado ao estresse oxidativo, que pode ter implicações profundas para as funções cognitivas. A Teoria dos Radicais Livres do Envelhecimento destaca o papel potencial do dano oxidativo acumulado no envelhecimento celular e no desenvolvimento de doenças relacionadas à idade. Esse desequilíbrio está ligado a alterações na reologia dos eritrócitos, contribuindo potencialmente para a redução do fluxo sanguíneo e o comprometimento da função cardiovascular. Devido à sua alta atividade metabólica e sensibilidade às condições oxidativas, o cérebro é particularmente vulnerável a esses efeitos. Propõe-se uma via integrativa que descreva uma cascata de interações desde o estresse psicológico até a redução das funções cognitivas. Nesse modelo, hipotetiza-se que o estresse psicológico contribua para alterações hemorreológicas que diminuem a disponibilidade de oxigênio e nutrientes para os tecidos neurais, impactando subsequentemente o desempenho cognitivo. Por fim, este manuscrito é uma revisão narrativa que apresenta um arcabouço conceitual resumido em uma figura integrativa, destacando a interação entre os mecanismos oxidativos, vasculares e hemorreológicos.

## INTRODUCTION

Psychological stress is a complex phenomenon associated with a series of reactions in the human body, including increased oxidative damage^1^. A vivid example of this link between stress and physical health can be observed in premenopausal women serving as caregivers for children with chronic illnesses^2^. Research has shown that individuals who reported greater perceived stress also had higher levels of oxidative stress and shorter telomeres, a marker of cellular aging^3^. Although this association is well established, the underlying mechanism remains uncertain.

Epidemiological research has shown that chronic stress might predispose an individual to an exaggerated cortisol response, potentially accelerating biological aging and contributing to the development of disorders such as depression^4,5^. Reactive oxygen species (ROS), which include radical and partially reduced non-radical oxygen molecules, are generated during metabolism through interrelated reactions and are associated with progressive damage to deoxyribonucleic acid (DNA), proteins, and lipids throughout an individual’s lifetime^6^.

The brain is particularly susceptible to oxidative stress due to the reuse of various reactive species to carry out multiple signaling functions. Due to the high lipid concentration and energy demand, the brain is vulnerable to degradation associated with free radical activity^7,8,9,10^.

In the cardiovascular context, several theories explain the normal aging process, including oxidative stress, free radical production, neuroendocrine alterations, and genetic predisposition^11^. These factors, which primarily affect myocytes and the intima-media layer of the arteries, contribute to increased ventricular and vascular stiffness, a phenomenon intrinsically linked to cardiovascular aging^12^. Studies have shown that ROS impair red blood cell function by reducing deformability and increasing aggregation^13^, consequently elevating blood viscosity (BV). Furthermore, Marioni et al.^14^ identified BV as a key determinant of cognitive functioning. One study showed a significant correlation between decreased cognitive function and increased BV, suggesting that high BV represents a potential biomarker to be investigated in future research^15^.

In this review, the aim was to underscore the physiological pathways underlying the relationship between psychological stress and cognitive deficits. Understanding how psychological stress affects cognitive function through oxidative stress and hemorheological changes in BV and cerebral blood flow (CBF) is an important focus for ongoing research. As highlighted in the literature, BV has the potential to modulate CBF, thereby impacting cognitive function and underscoring the clinical relevance of this axis. The rationale for this review is to clarify the relationships among psychological stress, oxidative pathways, and cardiovascular function, as well as their implications for cognitive function.

## NARRATIVE REVIEW

This manuscript is a narrative review aimed at conceptually integrating evidence linking psychological stress, oxidative and hemorheological mechanisms, CBF regulation, and cognitive performance. To construct this integrative model, a comprehensive literature review was conducted between January 2021 and November 2025. Relevant studies were identified through targeted searches in databases including PubMed/MEDLINE, Scopus, and Web of Science. The main search terms comprised combinations of ‘psychological stress’, ‘oxidative stress’, ‘blood viscosity’, ‘hemorheology’, ‘cerebral blood flow’, ‘endothelial dysfunction’, ‘nitric oxide’, and ‘cognition’. The search strategy focused on identifying mechanistic links between psychological stress, oxidative stress markers, and hemorheological parameters. The selection criteria prioritized peer-reviewed articles providing evidence on the impact of free radicals on vascular and neuronal health, the role of nitric oxide in endothelial function, and the subsequent effects of BV on CBF and cognition. Inclusion efforts focused on both human clinical trials and experimental animal models to provide a comprehensive physiological framework for the proposed integrative pathway.

### The proposed pathway

**Figure 1:**
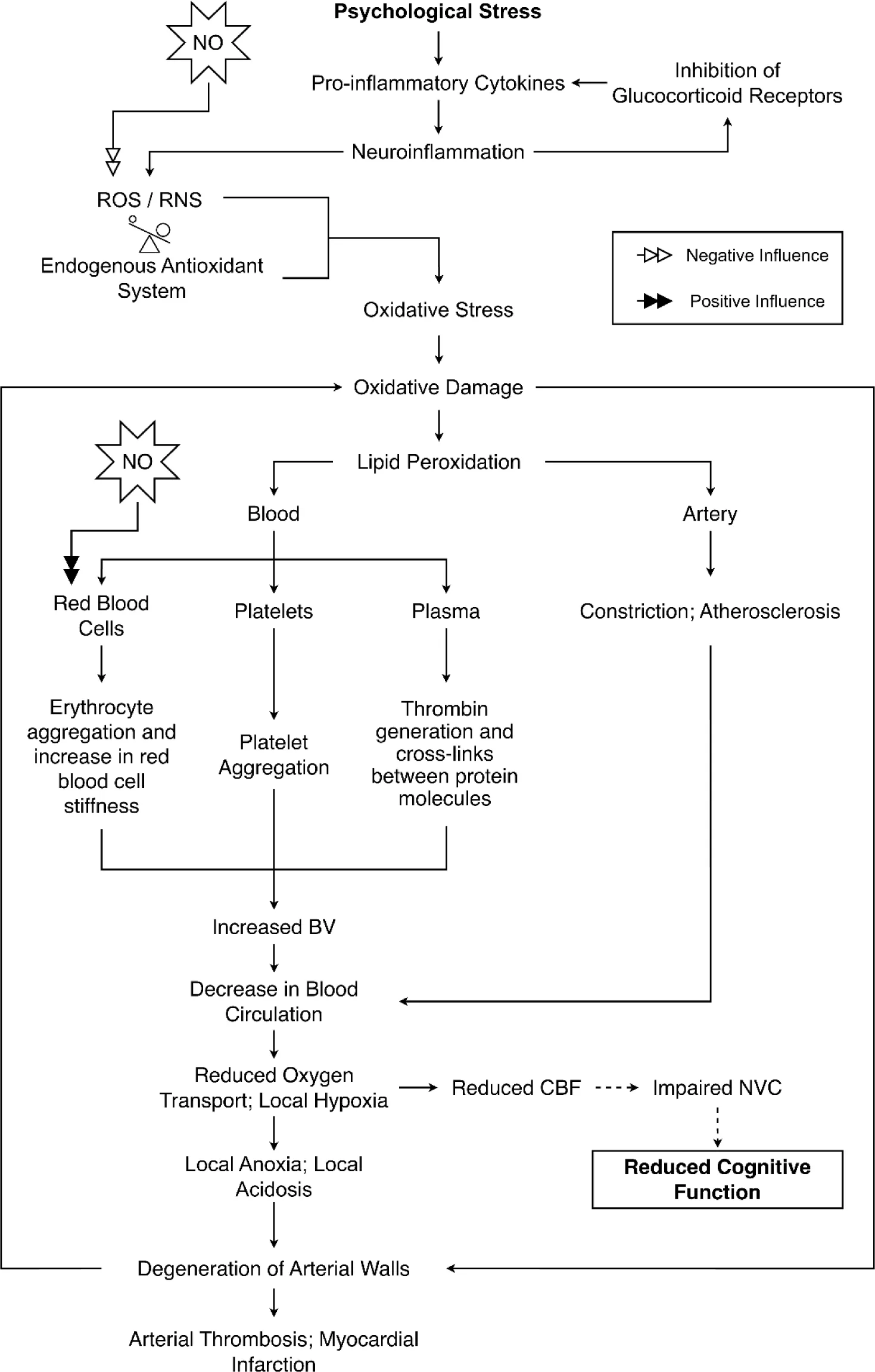
Integrative pathway linking psychological stress, oxidative damage, hemorheological properties, and cognitive function. Chronic psychological stress triggers neuroinflammation and disrupts the endogenous antioxidant system, leading to an overproduction of reactive oxygen and nitrogen species (ROS/RNS). This oxidative stress induces lipid peroxidation and reduces nitric oxide (NO) bioavailability, altering the cellular and plasmatic components of the blood. The resulting increase in blood viscosity (BV) impairs microcirculation, leading to local hypoxia and reduced cerebral blood flow (CBF). Consequently, this cascade is hypothesized to impair neurovascular coupling (NVC), ultimately contributing to reduced cognitive function. The diagram illustrates the dual role of NO: its essential physiological contribution to maintaining erythrocyte deformability and vascular tone (indicated as a positive influence), and its depletion through reactions with ROS, which yields the potent oxidant peroxynitrite (ONOO^−^), thereby exacerbating oxidative damage (indicated as a negative influence). Solid arrows represent established physiological pathways, whereas dashed arrows represent hypothesized inferential links in the stress-cognition axis.

As proposed in this review, oxidative stress associated with psychological stress may impact BV, neurovascular coupling (NVC), and CBF. Cognitive function is potentially affected by a decreased oxygen supply, mediated by hemorheological alterations. A pathway is suggested in Figure 1 that outlines a series of interactions associating psychological stress to diminished cognitive abilities. This figure represents an integrative hypothesis for the physiological mechanisms discussed herein. This pathway highlights the roles of neuroinflammation, free radicals (such as reactive oxygen and nitrogen species – ROS/RNS), the endogenous antioxidant system, and nitric oxide (NO) in oxidative stress.

Oxidative stress is associated with endothelial dysfunction, particularly through lipid peroxidation, which affects hemodynamics and further compromises CBF. Human cognitive functioning is closely tied to the body’s hemodynamic status. As illustrated in the proposed pathway, NO has a dual role. A negative influence arises when NO reacts with superoxide, leading to increased oxidation and the formation of peroxynitrite. On the other hand, NO exerts a positive influence by regulating vascular homeostasis, particularly by modulating erythrocyte deformability and bioavailability.

### Stress, the nervous system, and cognition

Psychological stress activates various physiological systems, including the autonomic nervous system and the hypothalamic-pituitary-adrenal (HPA) axis, resulting in the production and release of glucocorticoids. Due to their lipophilic properties, these steroids can diffuse through the blood-brain barrier and exert long-term effects on neural circuits involved in attention, learning, and memory^16,17^. Chronic stress, in particular, has been associated with brain atrophy and structural modifications. In the hippocampus, a region central to cognitive processing, glucocorticoids may impact neurogenesis, neuronal survival, and dendritic arborization, potentially contributing to the cognitive deficits observed in elderly individuals^18,19,20^.

The impact of stress on cognition depends on its intensity, duration, and magnitude^16^. While acute stress responses facilitate survival, severe or prolonged stressors can have deleterious effects on a broad range of cognitive functions. This process is closely linked to the interaction between hippocampal glucocorticoid receptors and pro-inflammatory cytokines. Elevated cytokine levels and functional glucocorticoid receptor resistance drive a vicious cycle that intensifies chronic neuroinflammation^21,22^. Such pro-inflammatory mechanisms are associated with exacerbated cellular stress and may represent an important mechanistic link between psychological distress and subsequent oxidative damage^23,24,25^.

### Oxidative stress and endogenous antioxidant system

To clarify the mechanisms discussed in this review, it is necessary to differentiate related concepts within this framework. Oxidative stress, also referred to as oxidative imbalance, describes a state in which the production of reactive species exceeds the capacity of antioxidant defense systems. This condition reflects a disruption of homeostasis between reactive species generation and endogenous antioxidant responses^26^. When sustained or severe, this state can lead to oxidative damage, characterized by the degradation of macromolecules such as DNA, increased lipid and protein oxidation, and the accumulation of dysfunctional mitochondria^27,28^.

When oxygen interacts with specific molecules, it can generate free radicals, which are highly reactive atoms or molecules with one or more unpaired electrons^9^. The terms reactive oxygen species (ROS) and reactive nitrogen species (RNS) refer to reactive radicals and nonradical derivatives of oxygen and nitrogen, respectively, which are produced by all aerobic cells^29^. Table 1 lists the primary types of cellular oxidants^30^.

**Table 1:** Examples of the various free radicals and other cellular oxidants.

Reactive species	Examples
ROS	Superoxide (O_2_˙^﹣^), hydroxyl radical (˙OH), hydrogen peroxide (H_2_O_2_), alkoxyl radical (RO˙), lipid alkoxyl (LO˙), peroxyl radical (RO_2_˙), ozone (O_3_), lipid peroxide (LOOH), singlet oxygen (^1^O_2_), hydroperoxyl radical (HO_2_·)
RCS	Hypochlorite ion (OCl^−^), nitryl chloride (NO_2_Cl)
RNS	Nitric oxide (NO), nitrous acid (HNO_2_), nitrosonium cation (NO^+^), nitrosyl anion (NO^−^), peroxynitrite (ONOO^−^), nitrogen dioxide (NO_2_), alkyl peroxynitrite (ROONO)
RSS	Thiyl radical (R-S˙), perthiyl radical (RSS˙)

Abbreviations: ROS, Reactive oxygen species; RCS, Reactive chlorine species; RNS, Reactive nitrogen species; RSS, Reactive sulfur species.

Source: Halliwell (2012)^30^.

The brain is particularly vulnerable to oxidative stress. This vulnerability is primarily due to its high concentration of polyunsaturated fatty acids, which are highly susceptible to lipid peroxidation. Additionally, glial cells can be a source of ROS synthesized by nicotinamide adenine dinucleotide phosphate (NADPH) oxidases. When combined, these factors can exacerbate and reinforce neuroinflammatory processes^28,29,30,31,32^.

To neutralize these free radicals and prevent oxidative damage, the body relies on endogenous antioxidant systems. In the context of cardiovascular and neural health, the coordinated action of three main enzymes regulates redox balance: superoxide dismutase (SOD), catalase (CAT), and glutathione peroxidase (GPx)^30,33,34^. The neutralization process begins with SODs, which regulate oxidative stress by transforming highly reactive superoxide radicals (O_2_^-^) into oxygen and hydrogen peroxide (H_2_O_2_)^35,36^. Subsequently, CAT and GPx work together to break down H₂O₂ into water and oxygen^37,38,39^. While these reactions occur throughout the body, GPx plays a prominent role in brain tissue because CAT activity in the brain is low and confined to peroxisomes, thereby allowing GPx to assume the primary role of eliminating excess H_2_O_2_^30,40^. High levels of oxidative stress can impair this delicate enzymatic homeostasis and have been associated with neurodegenerative and neuropsychological disorders^40^.

### Nitric oxide

NO is a gaseous signaling molecule involved in physiological processes, including vasodilation, neurotransmission, and immune regulation. In the vascular system, NO maintains homeostasis by enhancing blood flow and preventing thrombosis by reducing cell adhesion and inflammation in macrovessels^41,42^. Furthermore, it mitigates vessel wall remodeling and constriction while promoting angiogenesis in the microvasculature. In the central nervous system, NO plays a role in neural communication, although its involvement in pathological states depends on its concentration and cellular source^43^.

The biological effects of NO depend on its bioavailability and local redox conditions. Under oxidative stress, superoxide rapidly scavenges NO, reducing its vasodilatory and anti-inflammatory actions while promoting the formation of peroxynitrite (ONOO^−^), a potent oxidant that induces nitrative and nitrosative stress^40,42,44^. This pathway triggers post-translational alterations in proteins and oxidative modifications of lipids and DNA, which can disrupt normal cellular signaling and contribute to endothelial dysfunction and neuroinflammation.

### Oxidative damage-induced changes to cardiovascular tissues

Oxidative stress affects both the cardiovascular endothelium and erythrocytes. Endothelial cells regulate vascular tone, blood fluidity, inflammation, and neovascularization. These functions are highly susceptible to excess ROS and inflammation, often driven by cellular senescence and protein damage^45,46,47^. The resulting reduction in NO bioavailability contributes directly to endothelial dysfunction and apoptosis^48,49,50^.

Beyond the endothelium, oxidative stress severely affects red blood cells (erythrocytes)^51,52^. Erythrocytes rely on endogenous antioxidant defenses, such as CAT and GPx, to protect hemoglobin and membrane integrity^37,53,54^. Under conditions of ROS accumulation, these defenses are compromised, leading to the binding of partially oxygenated hemoglobin to the erythrocyte membrane. This structural change reduces erythrocyte deformability and impairs microcirculatory blood flow. Such alterations result in reduced oxygen delivery to tissues, fostering states of hypoxia and, in severe cases, hemolytic processes and potential cognitive dysfunction^51,53^.

### Blood viscosity

Blood, like all matter, has properties that affect its deformation and flow. Hemorheology encompasses the study of blood flow and deformation, as well as the cellular and plasma components, and the effects these have on the vascular network in which blood circulates, on adjacent tissues, and on foreign materials in circulation^55^. More specifically, plasma viscosity, hematocrit, erythrocyte deformability, and erythrocyte aggregation are the primary factors that influence BV, a physiological parameter that regulates vascular resistance. Changes in any of these factors alter tissue perfusion and vascular resistance to blood flow^56^. Thus, BV is implicated in the pathophysiology of conditions such as atherosclerosis, myocardial ischemia, stroke, and cognitive impairment^57^. This interaction between blood and the vascular wall at the vascular endothelial interface is present throughout the human body; malfunctions at this interface are often reflected in dysfunctions of the factors that characterize BV. For example, erythrocyte stiffness alters viscosity dynamics, particularly within the microvasculature, thereby impairing proper aggregation^58^. Furthermore, according to Beris et al.^59^, a 1% increase in hematocrit corresponds to a 4% increase in BV. Regarding plasma components, fibrinogen and other proteins affect plasma viscosity, which is influenced by cardiovascular risk factors such as arterial hypertension^51^.

Hemorheological parameters, including erythrocyte deformability, are strongly influenced by NO bioavailability and local redox status. Reductions in NO and oxidative damage to hemoglobin and membrane proteins impair erythrocyte deformability. Furthermore, studies by Nader et al.^56^ and Forconi and Gori^60^ suggest that endothelial NO production acts as a compensatory mechanism in response to shear stress, regulating BV. In vascular dysfunction, this adaptive response is compromised, leading to elevated BV, increased vascular resistance, and accelerated arterial aging, a cycle further exacerbated by oxidative cascades. According to Nemeth et al.^61^, excess ROS, such as superoxide, trigger chain reactions that result in oxidative damage, including lipid peroxidation; this process affects transmembrane proteins and is associated with damage to hemoglobin molecules. This cascade of reactions, which highlights the role of free radicals in hemorheological properties, was elucidated by Mo et al.^62^ and subsequently corroborated by other studies^13,59,61,63^.

### Cerebral blood flow

CBF, which is closely linked to BV, is essential for sustaining the constant supply of nutrients and oxygen to brain tissues. Its precise regulation prevents flow variations that could lead to ischemia or tissue injury^64^. The cerebral vasculature, particularly the endothelial cells, plays a fundamental role in this process. Cerebral endothelial cells are central to maintaining vascular tone, permeability, and neurovascular homeostasis^65,66^. Consequently, endothelial dysfunction is often associated with compromised CBF. Physiological plausibility suggests that sustained perfusion impairments may threaten neuronal health in regions with high metabolic demand^65,67^.

To ensure stable perfusion, the brain relies on three primary regulatory mechanisms. First, autoregulation maintains constant CBF despite fluctuations in systemic blood pressure. Second, chemoregulation triggers robust vascular responses to changes in arterial CO_2_ or O_2_ concentrations^68^. Finally, NVC matches local blood flow to neuronal activity, supporting cognitive function. NVC ensures that active brain regions required for cognitive tasks receive adequate metabolic support by providing rapid, localized increases in perfusion^64^. Dysfunction in this complex system of regulatory mechanisms, including endothelium-dependent responses to hemodynamic stimuli (e.g., shear stress) and metabolic needs, may increase the risk of neurological disorders and have long-term detrimental effects on brain health^68^. Further research is required to translate these physiological findings into disease models, particularly regarding the role of CBF regulation in stroke, atherosclerosis, and neurodegenerative diseases^67^.

### Cognition, cerebral blood flow, and blood viscosity

The brain receives approximately 20% of cardiac output despite representing only about 2% of total body mass. This high perfusion demand reflects its role in cognitive, sensory, motor, autonomic, and homeostatic functions^69^. Cognition depends on tightly regulated cerebrovascular control, perfusion, and metabolism; therefore, relatively stable CBF is important for neural activity and metabolic homeostasis^70,71^. Over the past two decades, studies have examined the relationship between CBF and cognitive function^15,70,71,72,73,74,75^, generally reporting associations between lower CBF and poorer cognitive performance. However, Shoemaker et al.^71^ observed that an approximately 31% decrease in CBF was accompanied by only a modest, roughly 7%, reduction in cognitive performance, suggesting partial compensatory mechanisms. Overall, current evidence supports an association between age-related changes in CBF regulation and cognitive function^74^, while the direct contribution of CBF dysregulation to cognitive decline remains to be clarified.

Other factors contributing to cognitive decline include arterial remodeling and endothelial dysfunction, which are associated with the early stages of vascular cognitive impairment. Vascular aging, characterized by atherosclerosis and arterial stiffness, is known to weaken the blood-brain barrier and interfere with NVC^75,76,77^. Furthermore, cognitive decline is linked to hemorheological alterations, such as blood hyperviscosity and elevated fibrinogen levels, which tend to impair perfusion at the microvascular level^78^, particularly in aging populations^51,59^.

Clinical evidence has suggested a relationship between BV and cognitive function; for example, in the study by Falvo et al.^79^, older individuals with a higher proportion of larger erythrocytes were more likely to have memory problems. In the study by Pathansali et al.^80^, a link was observed between elevated BV, elevated homocysteine levels, and cognitive impairment. To mitigate the effects of hemorheological dysregulation on cognition, studies have investigated experimental therapies targeting BV to improve cognitive function. In a study combining acupuncture and hyperbaric oxygen therapy, Zhang et al.^81^ found that blood rheology and cognitive function improved in patients. Santos et al.^15^ used Ginkgo biloba extract, which was associated with improved cerebral perfusion and reduced BV, thereby improving cognitive performance in the experimental group, whereas cognitive performance worsened in the control group.

However, it is important to note that these interventional findings, particularly regarding acupuncture and herbal extracts, remain largely exploratory and heterogeneous and require further validation through large-scale, standardized clinical trials. In the current literature, the impact of hemorheology on cognitive function remains underexplored; much of the literature focuses on CBF and its dysfunctions, which have been shown to influence cognitive function^14,59,76,77,82,83^.

To clarify the strength of the current findings and guide future research, the evidence supporting this integrative model can be categorized into direct clinical associations and inferential physiological pathways. Direct evidence comprises clinical studies in which specific hemorheological parameters, such as BV, erythrocyte aggregation, and deformability, correlate with cognitive performance scores. In contrast, inferential evidence is derived from the physiological framework in which hemorheological changes are hypothesized to impair CBF and NVC, subsequently leading to observed cognitive deficits.

In conclusion, this review presents an integrative conceptual model connecting psychological stress, oxidative stress, cardiovascular health, and cognitive function. The proposed framework suggests that chronic stress may influence oxidative stress, potentially affecting neurological and cardiovascular systems. Within this model, elevated ROS are associated with processes that may impair endothelial function, increase BV, and modify CBF, all of which relate to cognitive decline.

The evidence highlights the role of the endothelium in vascular homeostasis and its connection to brain function. Additionally, the correlation between BV and cognitive function suggests that hemorheological factors are relevant to understanding cognitive impairment in aging populations. Further research is needed to investigate the effects of blood rheology on cognitive function and to evaluate potential targeted interventions. Future studies should prioritize the isolation of confounding factors, such as systemic inflammation, hematocrit variations, and pre-existing vascular comorbidities. Addressing these variables will help clarify the role of blood rheology within the complex axis linking psychological stress to cognitive health and further elucidate the pathways connecting cardiovascular health, oxidative stress, and cognitive performance.

## Data Availability

No new data were generated or analyzed in this study.
